# Rethinking detection of pre-existing and intervening *Plasmodium* infections in malaria clinical trials

**DOI:** 10.3389/fimmu.2022.1003452

**Published:** 2022-09-20

**Authors:** Tonny J. Owalla, Dianna E. B. Hergott, Annette M. Seilie, Weston Staubus, Chris Chavtur, Ming Chang, James G. Kublin, Thomas G. Egwang, Sean C. Murphy

**Affiliations:** ^1^ Department of Immunology and Parasitology, Med Biotech Laboratories, Kampala, Uganda; ^2^ Department of Laboratory Medicine and Pathology, University of Washington, Seattle, WA, United States; ^3^ Department of Epidemiology, School of Public Health, University of Washington, Seattle, WA, United States; ^4^ Center for Emerging and Re-emerging Infectious Diseases, University of Washington, Seattle, WA, United States; ^5^ Department of Global Health, University of Washington, Seattle, WA, United States; ^6^ Seattle Malaria Clinical Trials Center, Vaccine and Infectious Disease Division, Fred Hutchinson Cancer Research Center, Seattle, WA, United States; ^7^ Department of Microbiology, University of Washington, Seattle, WA, United States

**Keywords:** *Plasmodium falciparum*, pre-existing infection, intervening infection, clinical trial, at-home DBS, 18S rRNA

## Abstract

Pre-existing and intervening low-density *Plasmodium* infections complicate the conduct of malaria clinical trials. These infections confound infection detection endpoints, and their immunological effects may detract from intended vaccine-induced immune responses. Historically, these infections were often unrecognized since infrequent and often analytically insensitive parasitological testing was performed before and during trials. Molecular diagnostics now permits their detection, but investigators must weigh the cost, complexity, and personnel demands on the study and the laboratory when scheduling such tests. This paper discusses the effect of pre-existing and intervening, low-density *Plasmodium* infections on malaria vaccine trial endpoints and the current methods employed for their infection detection. We review detection techniques, that until recently, provided a dearth of cost-effective strategies for detecting low density infections. A recently deployed, field-tested, simple, and cost-effective molecular diagnostic strategy for detecting pre-existing and intervening *Plasmodium* infections from dried blood spots (DBS) in malaria-endemic settings is discussed to inform new clinical trial designs. Strategies that combine sensitive molecular diagnostic techniques with convenient DBS collections and cost-effective pooling strategies may enable more thorough and informative infection monitoring in upcoming malaria clinical trials and epidemiological studies.

## Introduction

Clinical trials are critical for evaluating candidate malaria vaccines and drugs. Such trials are routinely conducted in malaria-endemic sites as field efficacy trials (1–5) and in both endemic and non-endemic sites as controlled human malaria infection (CHMI) studies (6–9). In all cases, it is generally accepted that the *Plasmodium* infection status of the participants is established at the time of trial enrollment. In malaria-endemic regions, participants may have been recently exposed to *Plasmodium* parasites, so it is possible that participants may be actively infected at the time of trial eligibility and enrollment assessments. Consequently, many studies are designed to start with anti-malarial drug treatment of some or all participants to eliminate any pre-existing *Plasmodium* parasites at the outset of the trial (4, 6, 7, 10, 11). In CHMI studies in non-endemic regions (8, 9) and field efficacy trials involving children 5-17 months in endemic settings (1–3, 5, 12), pre-treatment is not usually considered because participants are usually assumed not to have pre-existing *Plasmodium* infections. However, at least one pre-existing, low-density *Plasmodium falciparum* infection was encountered during screening and eligibility procedures at a U.S.-based non-endemic CHMI study site (S. Murphy, J Kublin, pers. comm.), which highlights the need for pre-enrollment testing worldwide. Pre-enrollment testing is a requirement for any non-endemic CHMI study intending to use a recently qualified *Plasmodium* 18S rRNA biomarker in lieu of thick blood smears (TBS) for detecting infections in such studies (13). Pre-enrollment testing has also been used in one CHMI study in an endemic region (14). On the other hand, following vaccination, most field studies in malaria endemic settings rely on passive case detection for endpoint efficacy assessments such as time to first infection or to first episode of clinical malaria (1, 5). Studies employing active case detection through weekly or monthly visits usually only collect a thick blood smear (TBS) if a participant reports a temperature of ≥37·5°C or history of fever and other malaria-related symptoms within the last 24 hours (2–4). Examples of the current field practices employed by investigators during the pre-enrollment, follow-up sampling and efficacy endpoint assessment are as shown in **Table 1**. It is clear that reliance on symptoms as well as weekly or monthly sampling and low sensitivity techniques may miss out on pre-existing and emerging *de novo* low density infections, which may confound vaccination efforts and ultimately affect efficacy estimates.

**Table 1:** Examples of clinical trial strategies for pre-vaccination treatment, follow-up sampling, and efficacy endpoint assessments.

Vaccine candidate	Clinical trial design	Pre-vaccination treatment? (if any)	Infection detection endpoint?	Follow-up during efficacy and infection detection?	Reference
RTS,S	Field trial at 11 African sites in children	None (enrolled infants and children 5-17 months)	Clinical malaria; severe malaria (TBS)	PCD for >18 months	(1)
R21	Field trial in Burkina Faso in children 5-17 months	None. Participants tested for malaria if fever ≥37·5°C.	Clinical malaria (TBS)	ACD monthly for 6 months plus PCD. TBS obtained if temperature ≥37·5°C or history of fever within the last 24 h.	(2)
SPf66	Field trial in The Gambia in children 5-11 months	Antimalarial treatment before first and third vaccination (SP)	Clinical malaria (TBS)	ACD twice weekly for 4.5 months plus PCD. TBS obtained if temperature ≥37·5°C or history of fever within the last 24 h.	(3)
DNA/MVA ME-TRAP	Field trial in The Gambia in children and adults	Antimalarial treatment prior to 3^rd^ dose of vaccination (SP)	Infection by TBS	ACD and weekly TBS for 11 weeks.	(4)
ChAd63 MVA ME-TRAP	Field trial in Burkina Faso in 5-17 months	None (enrolled infants and children 5-17 months)	First clinical malaria episode (RDT & TBS)	PCD and TBS if temperature ≥37·5°C or history of fever within the last 24 h	(5)
GMZ2	CHMI in adults in an endemic region (Gabon)	Antimalarial treatment prior to vaccination (clindamycin)	Infection by TBS & qRT-PCR	ACD for 6-35 days	(7)
PfSPZ	Phase 2 field trial in Kenya in children	None (enrolled children 5-12 months)	Clinical malaria and infection (TBS)	ACD (RDT) and PCD (TBS/qPCR) every two weeks for 12 months	(12)
PfSPZ CHMI	CHMI in adults in an endemic region (Kenya)	None prior to CHMI; tested for existing infection	Clinical malaria & qPCR (treated at ≥500 parasites/µL)	ACD (blood drawn twice per day from days 8-15 and once from days 16-22 post-CHMI)	(14)

ACD, active case detection; PCD, passive case detection; SP, sulfadoxine-pyrimethamine.

### What are the consequences of low-density pre-existing and intervening infections on measurement of parasitological efficacy endpoints in vaccine studies?

The presence or absence of *Plasmodium* parasites or of a parasite-derived biomarker are often used in studies designed to assess time to first infection or time to first clinical episode as efficacy endpoints. Such assessments depend on accurate identification of pre-existing *Plasmodium* infections at enrollment and during follow-up. However, definitive determination of the infected *vs*. uninfected baseline status of a participant can be difficult because a significant proportion of *Plasmodium* infections in endemic regions exist at low densities (15–17), which are often below the limit of detection (LoD) of standard field diagnostic tools such as TBS and rapid diagnostic tests (RDTs) (18, 19). Even in studies that use molecular tests, low-density infections may be missed because of the highly dynamic nature of the parasite – densities may be too low to be detected at the time of sampling. The inability to rule out pre-existing, low-density infections prior to vaccination and to detect their emergence during vaccination or in the subsequent efficacy assessment period may potentially confound trial outcomes and endpoint assessments. For example, undetected low-density infections could progress to higher density, detectable infections soon after enrollment – such infections would not be expected to be abrogated by vaccination with pre-erythrocytic vaccines and yet such pre-existing but undetectable infections could end up being counted as new infections in the study data, which could falsely reduce the calculated efficacy of a candidate vaccine product. Similarly, the inability to detect the emergence of low-density *de novo*/intervening infections after vaccination will extend the parasite detection time and has the potential to falsely amplify the calculated efficacy of the vaccine. Despite the likely influence of pre-existing and intervening low-density infections on vaccine efficacy, the significance and magnitude of such impacts is still poorly understood. For some vaccines, it is likely that vaccine efficacy is reduced when vaccinations are given concurrent with erythrocyte stage parasitemia, which was shown, for example, to reduce sporozoite-based vaccine efficacy in a CHMI model (9). However, there could be circumstances where the timing of an infection potentially enhance efficacy. For example, a study of the ChAd63/MVA ME-TRAP vaccine in Kenya resulted in 67% efficacy against field-acquired infections (20), which was higher than that observed in Senegal (21). *Post-hoc* analysis showed that the rate of *Plasmodium* infections during the vaccination period were much higher at the Kenyan sites than at the Senegalese sites (21), which could have modulated either anti-erythrocyte or liver-stage immunity. The complex effects of low *vs*. high parasitemias and other parasite, host, and environmental factors on the immune system must be evaluated in the future to develop and safeguard malaria vaccines.

### What are the consequences of pre-existing infections on measurement of immunological efficacy endpoints in vaccine studies?

Many studies have found that malaria vaccine efficacy is reduced in studies in endemic regions compared to efficacy against CHMI in non-endemic sites [discussed in (22)]. An extensive review of the contributing immunological, parasitological, vectorial, and environmental factors is beyond the scope of this paper. Instead, the following section highlights several recent clinical trial outcomes that demonstrate the consequences of pre-existing infections on vaccine study outcomes. First, a recent CHMI study at a non-endemic U.S. site showed that the administration of the second and third doses of *P. falciparum* sporozoite-based vaccine at 7-day intervals, concurrent with the emergence of low-density blood stage *Plasmodium* parasites (<20 estimated parasites/µL; TBS-negative) completely eliminated the otherwise high efficacy achieved when blood stage parasites were absent during vaccination with a two-fold higher dose of the same vaccine given at 5-day intervals (9). Second, field clinical trials of the recently WHO-approved RTS,S vaccine and other candidates reveal that immunity induced by candidate malaria vaccines is dependent on specific antibodies and requires an active response involving B cells and CD4^+^ T cells (1, 23, 24). However, active TBS-positive *Plasmodium* infections induced altered phenotypes and functionalities of dendritic cells (25), B cells (26) and T cells (27), causing a disruption in host immune responses to antigenic epitopes. Furthermore, natural exposure to persistent *P. falciparum* infections is known to increase the frequency of atypical memory B cell and CD4^+^ T cells expressing phenotypic markers of exhaustion (28). Therefore, pre-existing infections may alter immune reactivity by down-regulating vaccine-induced immune responses, providing a probable reason as to why promising results of experimental malaria vaccine candidates in non-endemic regions have often not been replicated in malaria-endemic areas (29). Whether low density infections are as impactful as higher density infections is currently unknown. Consequently, detection of pre-existing infections is imperative to control the confounding effects of such infections and to facilitate reliable and consistent interpretation of clinical trial results in different cohorts at different clinical sites under different transmission pressures.

## Low density *Plasmodium* infections – a frequent complicating factor worldwide

To our knowledge, there are no widely-accepted, standardized approaches for detection of pre-existing *Plasmodium* infections in malaria clinical trials in endemic regions. Nonetheless, the emerging literature suggests that these infections are common and therefore overlooked. A recent DBS study in a hyperendemic region of Uganda enrolled asymptomatic, RDT-negative persons to better understand the natural history of asymptomatic low-density infections (17). Amongst adults and children, 58% (76/130) of RDT-negative individuals had *Plasmodium* 18S rRNA detectable by quantitative Reverse Transcription Polymerase Chain Reaction (qRT-PCR) at some point during the 28-day collection period. This study is notable because DBS samples were self-collected using daily finger prick sampling to observe the dynamic behavior of these asymptomatic low-density infections. This study is discussed in a later section as well to provide a roadmap for improving infection detection in future endemic site clinical trials. Prevalent, dynamic asymptomatic low-density *Plasmodium* infections have also been reported by other investigators in different regions of malaria endemicity (15, 16, 30). In Mozambique, analysis of parasite densities collected at seven time points over 28 days in a cohort of asymptomatic men revealed that 81% were cumulatively parasite PCR positive by day 28 and that parasite densities continued to vary in individuals over that 28-day period (16). Similarly, a study in a low transmission setting in Vietnam also showed 32% of samples were PCR positive and that parasite densities in asymptomatic carriers oscillated over time (30). These low-density parasitemias would be considered to be pre-existing infections in malaria vaccine clinical trials. However, if testing is not planned throughout the study or if the testing modality is insufficiently sensitive, then such infections would go undetected and the consequence to the efficacy estimates of the experimental vaccine would be largely unknown.

## Diagnostic methods for detecting low-density infections in malaria clinical trials

Since 2010, WHO advised that all diagnoses of malaria febrile illnesses be accompanied by a confirmatory parasitological test (31), which could be microscopy, RDTs, or molecular testing. Such methods may detect low-density *Plasmodium* infections, albeit with different degrees of success. TBS, RDTs, and molecular tests can be conducted on capillary (fingerstick) blood or peripheral whole blood, which can be collected and stored as liquid blood or preserved as DBS. In clinical trials, Phase 1-2 studies typically schedule more frequent testing and use more analytically sensitive tests compared to Phase 3 studies. Test selection considerations also include the study population (infants, children, adults), the clinical and laboratory capabilities of the site, and assay costs. It should be noted that some malaria clinical trials have used clinical signs and symptoms of malaria as an eligibility criterion for enrollment (8) or as a trigger for diagnostic testing (2), but such approaches completely ignore the larger pool of pre-existing, low-density infections and *de novo* emerging intervening infections.

### TBS microscopy

Microscopic examination of TBS remains a standard method for the diagnosis of *Plasmodium* infections and for estimating parasite densities in most field studies. Briefly, preparation of a TBS involves spreading a drop of blood obtained *via* finger stick or venipuncture onto a clean, dry microscope slide. The TBS is allowed to dry, then erythrocytes are lysed and nuclei are stained with Giemsa stain for 10-30 minutes depending on the specific method. Parasite detection is performed under an oil-immersion light microscope at a total magnification of ~1000-fold. TBS microscopy allows for definitive identification of *Plasmodium* species by well-trained microscopists. The advantages and disadvantages of TBS have been extensively reviewed (32, 33). For our purposes, we will focus on three key factors: quality, scalability, and proximity. First, high-quality microscopy (like all high-quality laboratory testing) requires ongoing proficiency testing and quality control, which can be difficult since microscopy is more operator-dependent than the other testing methods. Even with high quality microscopy, the field-use LoD of TBS is relatively high at ~50-100 parasites/µL (34, 35) – this LoD would miss many asymptomatic and intervening low-density infections. The inability to detect low-density infections means that infected persons may be erroneously enrolled in studies or the subsequent emergence of such an infection can be delayed or missed during or after vaccination. Second, TBS microscopy is laborious and does not scale easily with increasing numbers of clinical trial samples. TBS may be required at frequent defined study time points, but also must be available on-demand for clinically-significant cases. The turnaround time for a small number of TBS is such that clinically-actionable data can be obtained within hours, but as the number of slides increases, it becomes harder to provide timely reporting. CHMI studies do not enroll extremely large numbers of participants, but daily TBS is at least usually required during periods when patent parasitemia is anticipated (8, 23, 24, 36). In contrast, field efficacy studies have less frequent sampling but usually enroll larger cohorts of participants (2, 7, 8, 10, 23, 37). Thus, in both studies, delivery of timely, high quality TBS results can be difficult. Nonetheless, TBS can be performed at or near the clinic. While proximity to the clinical site is critical for symptomatic case management, such proximity may be less important when monitoring and following up on low-density infections.

### RDTs

RDTs are lateral flow immunochromatographic tests that detect *Plasmodium* antigens in whole blood (usually histidine rich protein-2 (HRP2) for *P. falciparum* and lactate dehydrogenase (LDH) for all species). They have the advantages of ease of use, rapid turnaround time suitable for point-of-care or near point-of-care use, and therefore, deployability. However, the LoD for most marketed RDTs is ~200 parasites/µL (38), which would miss many pre-existing and intervening low-density infections. Newer ‘ultrasensitive’ RDTs (uRDT) have LoDs about 10-fold better than standard RDTs (39, 40), but these are not yet widely available. In addition to the high LoD, *P. falciparum* parasites with deletions in the HRP2-coding gene can lead to false negative RDT results (41, 42), which limits their use. RDTs may also remain positive following parasite clearance due to persistent antigenemia even in appropriately treated persons such that RDTs are not considered to be a test of cure. Finally, RDTs do not provide any quantitative assessment of parasite density, which is useful for modeling parasite growth and estimating the impact of partially protective vaccines through measures such as estimated liver burden. RDTs have not been widely used as an efficacy endpoint in malaria vaccine clinical trials, though some groups are beginning to assess RDT diagnostic performance against TBS and qPCR in some CHMI trials (43).

### Nucleic acid amplification tests

Over the past forty years, a wide variety of nucleic acid amplification tests (NAATs) have been developed for many infectious diseases including malaria. In simplest terms, NAATs generally involve a nucleic acid extraction step followed by an amplification/detection step using oligonucleotide-specific reagents. Methods include PCR, qRT-PCR, and nucleic acid-based sequence amplification, which have been reviewed previously (32). Methods vary with respect to the amount of blood sampled, the amount of extracted nucleic acid carried into the amplification step, the strategy for detection, the target gene(s) or RNA sequence(s), the choice of oligonucleotide-specific reagents, and the scale of testing. For the purposes of detecting low-density infections in clinical trials, we recommend that NAATs should only be considered for use if they can reliably detect infections at densities <1 parasite/µL. Some NAATs achieve even more sensitive LoDs in the 0.001-0.02 parasite/µL range. Such assays generally sample 0.05-1 mL of blood, a much greater volume than can be examined by TBS. Sensitive NAATs can detect blood stage infections 1-4 days before TBS [reviewed in (13)]. NAATs are also less operator dependent than TBS and more scalable than TBS or RDTs for monitoring low density infections. Because of their superiority over TBS and RDTs, a variety of NAATs have been used in CHMI trials at both non-endemic sites (8, 9, 36, 44) and endemic sites (14, 23). To provide proficiency testing across different malaria NAAT platforms, the World Health Organization has established a formal external quality assurance scheme for malaria NAAT laboratories (45, 46). One drawback to NAATs is the requirement for sophisticated instrumentation and staff training, which often leads to NAATs being performed at only centralized/reference laboratories. A potential technical drawback is that some NAATs can also detect gametocytes and produce positive results at low densities that cannot be adjudicated by microscopy. Detection of gametocytes may lead to exclusion at enrollment, and detection of potentially pre-existing gametocytes following vaccination may confound parasitological efficacy endpoints, especially if more convenient, less sensitive testing were used at enrollment. The influence of gametocytes on molecular diagnostic tests used for malaria vaccine efficacy endpoints requires additional study and consideration as these tests become more widely adopted. As the field advances, these considerations will need to be balanced to implement NAAT strategies that speed turnaround times, simplify clinical site scheduling and sampling, provide clear and actionable data, and save on human resources and financial costs without sacrificing quality.

## A strategy for more frequent, cost-effective testing to avoid clinical trial blind-spots

DBS collection is a convenient, minimally invasive blood collection technique that does not require a clinic or phlebotomist. DBS remain stable over a wide range of temperature and storage conditions, and thus allow retrospective analyses without sacrificing sample integrity. A recent meta-analysis determined that DBS were non-inferior to venous blood samples for qualitative detection of *Plasmodium* parasites across a variety of settings (47). However, DBS continue to be mainly used for sample collection in clinic and field settings by trained healthcare professionals. Nevertheless, DBS have been used successfully for self-collection of samples for a variety disease conditions such as HIV (48, 49), hepatitis (50), and diabetes (51).

Recently, an alternative and cost-effective sampling approach based on at-home DBS collection combined with pooled *Plasmodium* 18S rRNA qRT-PCR was determined to be feasible, well tolerated, cost-effective, analytically sensitive, and convenient for detecting low density infections in asymptomatic adults and children in an endemic area (17). This feasibility study of daily at-home DBS collection was conducted in 130 (100 adults and 30 children) community members in a rural, malaria-endemic setting in Uganda (17). In this study, participants were minimally trained in DBS collection by study staff at enrollment and were supplied with DBS collection packages for at-home use for the subsequent six days. DBS were returned by participants to the clinic on the seventh day and retraining was conducted if necessary. Thereafter, each week, participants were provided with all materials to collect daily at-home DBS until the following week and this was repeated until day 28. Compliance with at-home DBS collection was extremely high, with 85% of participants collecting all DBS over the 28-day period. Only five (4%) participants withdrew from the study early due to pain or inconvenience of the collection procedures - details about the study are recently published (17). Accuracy of the at-home collected DBS as a parasite detection tool for low-density infections was also assessed using a recently adapted pooled qRT-PCR strategy (52). The method involved conducting initial DBS runs using within-participant pools of up to 10 samples per pool (equal to 10 daily DBS collections per pool). If the pool was negative, all samples were reported as negative. If the pool was positive, samples were deconvoluted and re-run individually. DBS pooling reduced costs associated with testing individual qRT-PCR negative samples, and qRT-PCR provided highly sensitive detection of parasite 18S rRNA biomarker. The feasibility of at-home, self-collected DBS in rural settings could improve the ability to conduct surveillance studies and trial follow-up. Additional data from this study will soon be forthcoming to share the prevalence and complexity of the asymptomatic infections seen in these participants (D. Hergott, S. Murphy, pers. comm.).

**Figure 1:**
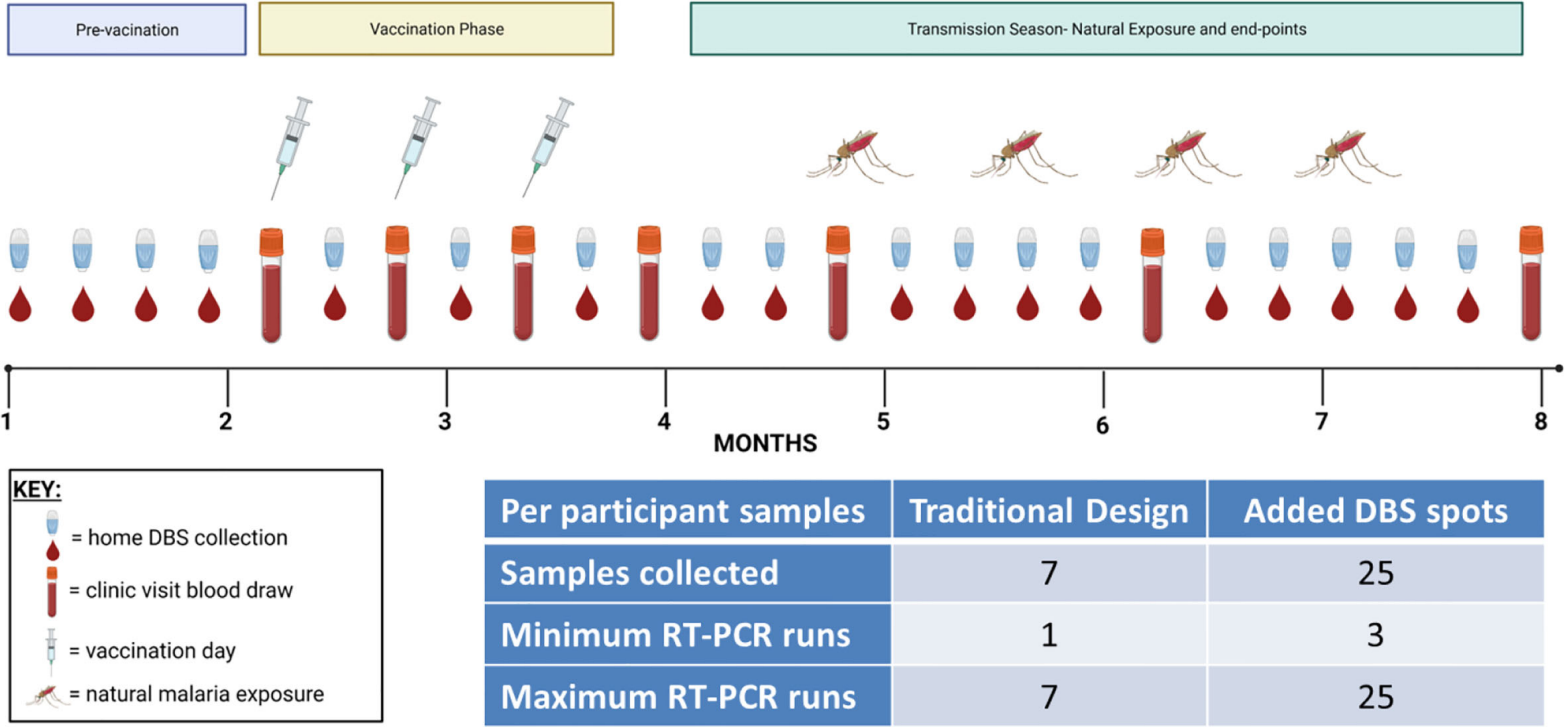
Proposed testing strategy for more frequent DBS collections with pooled qRT-PCR. In this theoretical vaccine clinical trial scenario, enrollment and vaccination take place over the first four months of the study followed by a four-month efficacy phase during the transmission season as shown. In addition to the typical whole venous blood collections (test tube icons), more comprehensive testing can be achieved by adding repeated DBS collections during the intervening time periods (small drop of blood icons). These DBS collections could be at-home or in the clinic as needed. The number of collections could be adjusted to a daily frequency or to less frequent collection as needed. The inset table shows the number of samples collected if only venous blood was specified (‘Traditional Design’) or if venous blood and DBS were collected (‘Added DBS spots’) and then calculates the minimum and maximum number of qRT-PCR tests that would need to be tested to determine each participant’s infection status; this calculation assumes a first qRT-PCR pool size of n = 10. The minimum number of runs would occur if all three pools were negative, whereas the maximum number of runs would occur if all three pools were positive and required deconvolution.

Malaria vaccine clinical trials in endemic regions usually involve periodic follow-up for months during and after vaccination. Study designs are intended to be long enough to capture a sufficient number of infections in the community to render a verdict regarding vaccine efficacy between two or more groups, and sampling is intended to be frequent enough so as to not miss an infection that could come and go between visits. However, participants are usually not sampled more than once a week and sometimes only once a month depending on the number of participants and the study (**Table 1**). Furthermore, in many studies, there is little or no infection detection monitoring during the vaccination period. These untested periods leave blind-spots in the study data that could potentially help to explain trial outcomes. Low-cost, at-home DBS collections with pooled qRT-PCR testing provide a way to comprehensively assess infection status before and during a study to avoid such blind-spots (**Figure 1**). This strategy reduces the number of clinic visits per participant, and pooled sample analysis also reduces the number of qRT-PCR runs per participant, thereby reducing cost. DBS can be collected before and throughout a study and delivered to the clinic site on a convenient basis once a week. The frequency of DBS collection, delivery, and testing for a given trial site would be informed by existing knowledge of the site’s seasonality and intensity of transmission. Home-collected samples could even potentially be mailed to a coordinating laboratory or picked up by village health workers or other Ministry of Health networks with access to the community. This approach would save time, human resources, and money associated with large scale and frequent sample collection and analysis. While this study was conducted using qRT-PCR, it is possible that other NAAT methods could also be similarly used with this overall strategy, provided that the assay LoD is sufficiently sensitive to detect pre-existing and intervening low-density infections at the pooled sample step.

The DBS testing described herein would not need to be done immediately after collection because this approach would be restricted to monitoring of low-density asymptomatic persons. Clinical trial sites would need to continue to provide TBS or RDTs to manage acutely-ill participants and initiate treatment as needed. From an ethical perspective, there is no current WHO mandate to treat asymptomatically-low density infected persons despite the known frequency of this type of infection throughout malaria-endemic parts of the world. If there was a long interval between collection and testing, the results may not be actionable for an individual participant. If the testing was conducted with a shorter turnaround time, it may be possible to relay actionable information back to clinic sites to inform treatment of participants. In addition to providing clear and comprehensive vaccine efficacy data, this infection status data could also help to better understand local prevalence and transmission characteristics. This sort of testing strategy could also be employed in large scale surveillance and longitudinal cohort studies over an even wider range.

## Conclusions

Malaria clinical trials that incorporate at-home DBS sample collection coupled with pooled qRT-PCR sample analysis may be better able to conveniently and cost-effectively detect pre-existing and intervening low-density *Plasmodium* infections in study participants. This rich information could provide valuable insights that will help us better understand why vaccines are efficacious in some participants and settings but not others, which could accelerate the development of new and improved malaria vaccines for the world.

## Data availability statement

The original contributions presented in the study are included in the article/supplementary material. Further inquiries can be directed to the corresponding author.

## Ethics statement

The perspective presented here is based in part on our recently completed study that was approved by the National HIV/AIDS Research Committee of the Uganda National Council for Science and Technology (UNCST) (Approval #: ARC 228) as well as the University of Washington Institutional Review Board (STUDY00009434). No potentially identifiable human data is presented in this article.

## Author contributions

TO, SM, JK, and TE conceived the idea. TO drafted the paper. DH, AS, WS, CC, and MC reviewed the first draft. All authors contributed to the article and approved the submitted version.

## Funding

OPP1212966/INV-009313 (SM), NIH R21AI146763 (SM).

## Conflict of interest

The authors declare that the research was conducted in the absence of any commercial or financial relationships that could be construed as a potential conflict of interest.

## Publisher’s note

All claims expressed in this article are solely those of the authors and do not necessarily represent those of their affiliated organizations, or those of the publisher, the editors and the reviewers. Any product that may be evaluated in this article, or claim that may be made by its manufacturer, is not guaranteed or endorsed by the publisher.

## Abbreviations

DBS, Dried blood spot; TBS, Thick blood smear; PCR; polymerase chain reaction; qRT-PCR, Quantitative real-time polymerase chain reaction; NAAT; nucleic acid amplification test; ACD; active case detection; PCD; passive case detection.
